# Cellular plasticity as a therapeutic vulnerability: HNF4α is a key target in lung adenocarcinoma

**DOI:** 10.1172/JCI207473

**Published:** 2026-07-01

**Authors:** Raymond Ho, Jason C. Mills

**Affiliations:** 1 Department of Molecular and Precision Medicine, Penn State College of Medicine, Hershey, Pennsylvania, USA.; 2 Medical Scientist Training Program, Baylor College of Medicine, Houston, Texas, USA.

## Abstract

Cells use plasticity programs to change lineages, which aids in tissue regeneration and remodeling but also allows aberrant cells to become cancerous and escape therapy. For example, tumor cells in invasive mucinous adenocarcinoma (IMA) emerge from lung epithelial cells by a plasticity program that reprograms them into gastric epithelium–like cells. In this issue of the *JCI*, Dadzie et al. show that hepatocyte nuclear factor 4 α (HNF4α) promotes gastric identity in lung epithelial cells via a mechanism involving restriction of FOXA1 and FOXA2 transcription factors to gastric gene enhancer loci. HNF4α also promotes resistance to KRAS inhibition by increasing nuclear factor erythroid 2–related factor 2 (NRF2) activity. These findings may advance therapeutic avenues in IMA.

## Lung adenocarcinoma exhibits high lineage plasticity

Cellular plasticity is a hallmark of cancer because cancer cells can use plasticity programs to change phenotypes or differentiation states to resist therapies (1). In a specific subtype of lung cancer, invasive mucinous adenocarcinoma (IMA) of the lung, pulmonary epithelial cells can assume gastric epithelial identity because they lose the homeobox transcription factor NK2 homeobox 1 (NKX2-1, also known as TTF-1), a key specifier of normal lung epithelial development (2). Additionally, the majority of IMAs highly express *KRAS* mutations (3), serving as a potential target in treatment considerations. IMAs are also characterized by expression of FOXA family transcription factors, which function as so-called “pioneer” transcription factors that help open chromatin to make it more accessible for other transcriptional modulators. In normal lung epithelial cells, NKX2-1 and FOXA1 or FOXA2 are often expressed in the same cells and coregulate cassettes of genes; however, FOXA1 and FOXA2 (FOXA1/2) transcription factors are also important for epithelial cells of other organs like the stomach, intestines, and liver. It has been unclear how FOXA1/2 alone could cause IMAs to acquire gastric differentiation.

## HNF4α drives plasticity and growth in IMA

**Figure 1:**
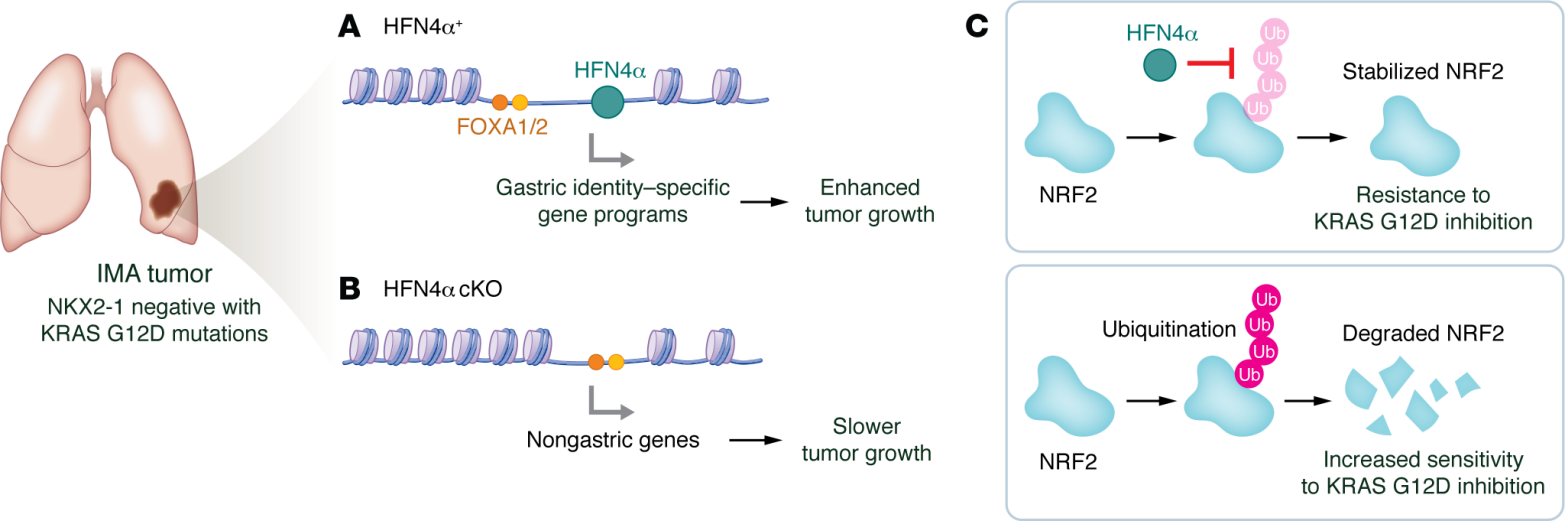
HNF4α drives plasticity and resistance to KRAS inhibition in IMA. Dadzie et al. (9) identified mechanisms governing plasticity and therapeutic resistance in IMA, a subtype of lung cancer characterized by loss of NKX2-1 and KRAS mutations. (**A**) They have shown that HNF4α cooperates with the pioneer transcription factors FOXA1/2 to promote IMA growth and gastric identity. (**B**) In the absence of HNF4α, FOXA1/2 bind to nongastric gene loci, and IMA growth is slowed. cKO, conditional KO. (**C**) Dadzie et al. also found that HNF4α stabilizes the NRF2 protein through regulation of its ubiquitination, which promotes resistance to KRAS inhibition. When NRF2 protein expression is low, IMA sensitivity to such therapy is increased.

Like FOXA1/2, hepatocyte nuclear factor 4 α (HNF4α) regulates gastric, intestinal, and hepatic cell differentiation (4, 5). Moreover, it has been implicated in many different cancers as both an oncogene and tumor suppressor (6). Interestingly, unlike NKX2-1 and FOXA1/2, HNF4α is not expressed in normal lung tissue but is expressed in IMA (7). In previous work, Fort et al. demonstrated that when functional NKX2-1 is present in a different, non-IMA subtype of lung cancer, HNF4α regulates a mixed gastric and pulmonary cell identity (8). However, in this issue of the *JCI*, Dadzie et al. showed that in IMA, the loss of NKX2-1 coupled with expression of HNF4α steered these cells specifically to the gastric phenotype (9). Using mouse and organoid models of IMA with or without HNF4α, the authors also found that HNF4α promoted tumor growth in IMA (Figure 1A). Thus, loss of HNF4α caused the expected drift away from gastric phenotype but also slowed tumor growth.

## HNF4α restricts FOXA1/2 activity to gastric loci

As mentioned above, previous work has shown that the pioneer transcription factors FOXA1/2 contribute to the IMA gastric identity (2). FOXA1/2 are known to work together with HNF4α, particularly in the liver (10), but whether they cooperate in IMA is unclear. By integrating data from ChIP-seq, assay for transposase-accessible chromatin using sequencing (ATAC-seq), and bulk RNA-seq experiments, Dadzie et al. provided strong evidence for a mechanism of reprogramming within the IMA-generating cells that features sequential cooperation between FOXA1/2 and HNF4α. Specifically, FOXA1/2 first establishes a permissive chromatin state (i.e., it “opens” genes) that allows HNF4α to bind and induce gastric-specifying genes. Loss of HNF4α caused FOXA1/2 to bind nongastric gene loci (Figure 1B). Thus, a likely mechanism is that FOXA1/2 opens gastric gene chromatin, allowing HNF4α to bind, and HNF4α thereafter also serves to enhance or reinforce FOXA1/2 binding to these same genes, preventing FOXA1/2 from “wandering” to other chromatin regions. These findings have therapeutic implications, because they clarify that HNF4α drives plasticity in IMA and, more important, maintains tumor growth that resists therapy.

## Targeting the HNF4α/NRF2 axis in IMA

Dadzie et al. also identified one mechanism that HNF4α may use in the context of IMA to drive tumor growth. The authors found that HNF4α maintained protein stability of the oxidative stress–regulating transcription factor nuclear factor erythroid 2–related factor 2 (NRF2) (11). By pharmacologically and genetically manipulating NRF2 abundance in IMA organoid models, they showed that organoid sensitivity to KRAS-inhibiting drugs was greatly improved when NRF2 was less abundant (Figure 1C). Thus, NRF2 might be targeted to decrease the effects of HNF4α in IMA without further affecting plasticity (as NRF2 has no known role in cell differentiation or cell identity).

## Origins of cellular plasticity

Cellular plasticity has been observed for over a century (12). It has been known almost as long that some adult tissues have dedicated stem cells that can give rise to the tissue’s usual cell lineages in tissue damage. Some have also implicated stem cells in plasticity, as when new, nonresident cell lineages emerge in a tissue (e.g., in the histopathological lesion known as metaplasia) (13). Metaplasias, and the tumors that can form from metaplasia, have been speculated to be fueled by stem cells changing their lineage and producing progeny that are ectopic for the tissue (14). It has also been proposed that such aberrant, lineage confusion of stem-like activity fuels established cancers, propelling study for the past couple of decades of so-called “cancer stem cells” (15). However, it should be noted that many, if not most, adult organs in which cancer arises do not have a dedicated population of stem cells. In those tissues, injury and inflammation induce cellular plasticity in mature cells such that they reprogram into progenitors. Progenitors derived from differentiated cells may be at particular risk for genomic damage due to erroneous DNA repair mechanisms and multiple other factors that make them more prone to potential cancer development than dedicated stem cells (16).

The reprogramming and recruitment of mature cells into stem cells may occur by an evolutionarily conserved program called paligenosis (17). Paligenosis occurs in three sequential stages: downscaling of the cell through autophagy, expression of embryonic/metaplasia genes, and cell-cycle reentry. Insults such as injury or infection can damage tissue, triggering paligenosis of differentiated cells to divide and repair the damage. Paligenosis can be viewed as the way multicellular organisms can recruit — and license — new stem cells from differentiated tissue. The licensing, which involves the tumor suppressor p53, may be critical, given the risk of genomic damage in differentiated cells. It has been proposed that cancers that arise from cells that initiate paligenosis but then continue to grow abnormally, independent of the initial tissue damage, may continue to divide using aberrant paligenosis mechanisms. It is uncertain how tumors like IMA develop and whether plasticity events like acquiring a gastric phenotype and avoiding therapy involve paligenosis, but clearly the field is ripe for more study on plasticity in cancer, in particular on the transcriptional and epigenetic reprogramming events like those observed in IMA (18).

Ultimately, in this study, Dadzie et al. have identified that HNF4α is a key driver of cellular plasticity in IMA, cooperating with FOXA1/2 to make epigenetic changes that give rise to gastric identity in the lung. They further uncovered an HNF4α/NRF2 axis in IMA that provides more options for designing targeted therapies. This work offers important insight into how diseases that depend on cellular plasticity can be treated.

## Conflict of interest

The authors have declared that no conflict of interest exists.

## Funding support

This work is the result of NIH funding, in whole or in part, and is subject to the NIH Public Access Policy. Through acceptance of this federal funding, the NIH has been given a right to make the work publicly available in PubMed Central.

- NIH grants R01DK094989, R01DK105129, and R01DK134531 (to JCM).

## Version 1 07/01/2026

Electronic publication
